# Lithontripteur Greatly Improved

**Published:** 1829-01-01

**Authors:** 


### XVIII.

### LITHONTRIPTEUR.

Mr. Zanabi Pecchioli, an eminent young surgeon, charged by the Grand Duke of Tuscany to observe the actual state of surgery in various countries, has made a great improvement on, or rather he has added a new and important principle to, the lithontriptic instruments invented by Messrs. Leroy d'Etoile, Civiale, Amusat, Hourteloupe, &c. &c. We have had a recent opportunity of examining Mr. Pecchioli's instrument, and seeing him work it on different calculi—not, of course, in the living body. We would say that its superiority over the instruments of the gentlemen abovenamed, is threefold. In the first place, it combines the principles of each of the others, the drills and other parts of their machinery being rendered completely available in Mr. P.'s apparatus. In the second place, the spring, or ressort, by which the drill or perforator is made to bear on the calculus, and which cannot, in the other instruments, be made to vary in force, is superseded by the construction of the pulley, which enables the operator to modify, vary, augment, or diminish, at pleasure, the force used—and that by his own hand. This we conceive to be a very important improvement. But the third modification is the most important of all. The perforator or drill, in Mr. P.'s lithontripteur, can, at any period of the operation, be converted into a kind of trephine, varying in the diameter of its circular movements from the smallest circle up to one of 18 lines in diameter, at the operator's will—and thus becoming capable of grinding down the calculus by a series of gyrations equal in extent to the grasp of the pincers or tenacula, instead of boring holes, and shifting the instrument for each perforation. By this operation, a considerable portion of stone may be ground down by a single sitting; and the danger of large and irregular fragments being scattered about in the bladder, when the calculus is broken after many perforations, according to the methods of Leroy d'Etoile and Civiale, is avoided.

Sir Astley Cooper, Mr. Travers, Mr. Key, and many other distinguished surgeons, have compared Mr. Pecchioli's apparatus with that of M. Civiale's; and, without vouching for the general success of the lithontriptic process, they have no hesitation in acknowledging the great ingenuity of Mr. P.'s instruments.

P. S. When Sir Astley Cooper was in Paris last month, he went to see M. Civiale operate with the lithontripteur. The subject was not one of the best for any operation. M. Civiale threw in half a pint of tepid water—introduced the lithontriptor with the greatest ease, seized the stone, drilled it, and then crushed it, all in the space of about seven or eight minutes. The operation over, the man discharged the water which had been injected, quite turbid with the sawings of the stone, and when poured off, disclosing numerous fragments that had come away with the first evacuation of the bladder after the operation. Sir Astley was quite astonished at the facility with which the whole was performed by M. Civiale. We apprehend that this operation *must* become popular in the hands of a *few* expert surgeons; but we do not suppose it will ever become general among surgeons at large.

